# Health-Related Quality of Life Among HIV-Infected Children and Its Association With Socio-Demographic, Clinical and Nutritional Variables: A Comparative Approach

**DOI:** 10.7759/cureus.25222

**Published:** 2022-05-22

**Authors:** Chinyere G Ogbonna-Nwosu, Kenechukwu K Iloh, Justus U Onu, Ifeanyi F Nwosu, Ngozi Ibeziako, Nnamdi Onyire, Dorathy C Obu, Chukwunonso A Nwosu, Nneka C Ezeudemba, Cynthia U Ifejika

**Affiliations:** 1 Pediatrics and Child Health, Alex Ekwueme Federal University Teaching Hospital, Abakaliki, NGA; 2 Pediatrics and Child Health, University of Nigeria, Enugu, NGA; 3 Psychiatry, Nnamdi Azikiwe University, Nnewi, NGA; 4 Child and Adolescent Mental Health, Federal Neuropsychiatric Hospital, Enugu, NGA; 5 Internal Medicine, Maimonides Medical Center, Brooklyn, USA; 6 Epidemiology and Public Health, Kaonic Research LLC, Gaithersburg, USA; 7 Infectious Disease, Medical University of Silesia, Katowice, POL; 8 Hematology and Medical Oncology, Emory University Winship Cancer Institute, Atlanta, USA

**Keywords:** clinical and nutritional variables, socio-demographic, caregivers' burden, hiv infection, health-related quality of life

## Abstract

Background: The human immunodeficiency virus (HIV) infection and its treatment impact the child’s life as well as that of their caregivers. As therapeutic advances are made in the field, improved survival has shifted the focus from morbidity and mortality to quality of life. This study aims to compare the health-related quality of life (HRQoL) of children living with HIV in comparison with an HIV-negative control group and determine its relationship with socio-demographic, clinical, and nutritional variables.

Method: This was a multi-center cross-sectional comparative study involving 274 participants (137 per group) carried out in tertiary and secondary level healthcare facilities in Nigeria. Socio-demographic, clinical, and nutritional variables were obtained using a researcher-designed data collection sheet. HRQoL was measured using the Paediatrics Quality of Life Inventory (PedsQL 4.0), while caregivers’ burden was assessed using the Zarit-Burden Interview (ZBI). A comparison of the health-related quality of life of the cases and the control group was done using an independent t-test. The predictors of HRQoL among the cases were measured using multivariate stepwise linear regression analysis.

Result: The overall health-related quality of life of HIV-infected children and those of the HIV-negative control group were similar. However, there was a significant difference in the school and psychosocial functioning domains between the two groups with HIV-positive children scoring lower in these domains. For HIV-positive children, being from upper social class (p = 0.01, R^2 ^= 0.098), male gender (p = 0.005, R^2 ^= 0.063), higher scores in the caregiver burden scale (p = 0.009, R^2 ^= 0.150) and more disease severity (p < 0.001, R^2 ^= 0.321) were significant predictors of lower health-related quality of life.

Conclusion: The findings of this study show that the overall health-related quality of life of HIV-positive children was similar to that of age- and sex-matched HIV-negative control group. This finding gives clinicians some optimism that with adequate treatment, HIV-positive children will have better outcomes not only in mortality but in psychosocial variables such as quality of life. In addition, the finding on the relationship between caregiver burden and HRQoL underscores the need to focus on family-based interventions to improve the burden of caregiving on family members involved in the care of HIV-positive children.

## Introduction

The human immunodeficiency virus (HIV) infection is a viral infection that attacks the body’s immune system and can lead to acquired immunodeficiency syndrome (AIDS) if not treated [1]. Currently, there is no cure for HIV infection but with antiretroviral drugs HIV infection can be significantly controlled [2]. Nigeria has the second-largest HIV epidemic in the world with about 3.1 million people living with HIV as of 2017 [3]; 220,000 of these were under 15 years of age [3].

With widespread availability and accessibility of antiretroviral therapy (ART), most HIV-infected children are now living long after the diagnosis, and HIV infection is now considered a chronic disease requiring long-term care [4]. Interventions in pediatric HIV/AIDS had focused on the medical treatment of the disease, with little emphasis on the long-term psychosocial issues and adjustments [5]. In addition, with the advances in therapeutics of HIV infections in children, measurement of disease outcomes evolved from morbidity and mortality to years lived with disability and quality of life [6-9].

Quality of Life has been defined as an individual’s perception of their position in life in the context of the culture and value systems in which they live and in relation to their goals, expectations, standards, and concerns [10]. In other words, Health-Related Quality of Life (HRQoL) is an individual’s perception of their quality of life in relation to health, disease, or treatment [10]. Several studies on the HRQoL of children with HIV infection have shown equivocal findings [4,6,11-14]. While some authors found significantly lower quality of life among HIV-infected children when compared to healthy control [11,12], others found no significant associations [4,9,13,14]. With regards to predictors of HRQoL, there is some evidence for some socio-demographic variables such as socioeconomic status [15,16], clinical factors such as being on antiretroviral medications [6,17], disease severity [4,17], and nutritional indices predicting health-related quality of life [18-20]. However, the literature is sparse with regards to the association of caregiver burden with the health-related quality of life of these children.

The aforementioned research evidence on HRQoL in children with HIV infection was in the Western and some Asian countries; however, this may not be the case in most African countries. Nigeria, the most populous nation on the continent has a dearth of studies on the quality of life of children living with HIV infections, though, modest research efforts were made in the adult population [8,9]. There is a need to study the HRQoL of children in Africa and its determinants, due to the following reasons: firstly, the socio-cultural determinants of health indices vary widely across continents and may affect disease outcomes; secondly, there is a need to contribute to the African perspective in the global meta-analysis of studies in health-related quality of children with HIV-infections. Based on the above, the following research questions became pertinent: firstly, Is the HRQoL of children living with HIV infection significantly different from the age- and sex-matched HIV-negative control? Secondly, are socio-demographic, disease severity, nutritional factors, and caregiver burden associated with health-related quality of life of children living with HIV infection?

## Materials and methods

Study design and setting

The study was a comparative cross-sectional study conducted in two hospitals in Abakaliki, Ebonyi State, South-Eastern Nigeria. The Pediatric HIV clinic and children's outpatient department of the Federal Teaching Hospital Abakaliki and the Mile Four Catholic Hospital Abakaliki, Ebonyi State, Nigeria were the study centers. The two hospitals were chosen based on the level of care provided; while the teaching hospital is the only tertiary care hospital in the state providing care for children living with HIV/AIDS, Mile Four hospital is a secondary care hospital with the largest number of pediatric HIV/AIDS clients in the state. These two health care facilities provided participants from the various background that was reflective of the various social cadres in Nigeria. While the patient population in the teaching hospital was primarily civil servants and middle class, that of the Mile Four hospital were mostly subsistence farmers and of lower socioeconomic class. 

Participants/sample selection

Subjects (HIV-positive children/Caregivers)

Study subjects (patient-caregiver pair) were recruited using systematic random sampling from a pool of clinic attendees per clinic day, with the sampling interval calculated from the total number of attendees in the age bracket present at the clinic and the number that the researchers intended to recruit per clinic day. HIV-positive children and their caregivers were the subjects of this study. The inclusion criteria were being an HIV-positive child aged 5-18 years, having been in care for at least one year, and having no other chronic medical condition. The caregivers, on the other hand, must have lived with the child for at least one year and were directly involved in the child’s care. A total of 140 HIV-positive children were approached, one declined participation in the study, and two were acutely ill with febrile illness at the time of the study. Out of the 137 that agreed to participate in the study, five children did not come with their primary caregivers. The primary caregivers of these five children were contacted by phone, a clinic appointment was fixed, and the patients and their caregivers were interviewed.

In computing the required sample size, the formula for comparing two means in two independent study samples was used [21]. A total of 137 patient/caregiver pairs per arm was arrived at, making it a total of 274 patient/caregiver pairs using 95% confidence interval and 80% power.

The control group (HIV-negative children/Caregivers)

The control group was age- and sex-matched HIV-negative children attending children's out-patient clinics for minor ailments or medical reports and their caregivers were recruited using a convenience sampling technique. The selected controls were approached individually by one of the researchers and consent was obtained from the caregiver and assent from the children aged seven years and above. HIV counseling and testing were offered to selected controls according to the National guidelines on HIV counseling and testing [22]. The control group had similar exclusion criteria to the subjects.

Ethical consideration

Approval for this study was obtained from the Ethics and Research Committee of the Federal Teaching Hospital Abakaliki and the Mile Four Hospital Abakaliki, with approval numbers REC Approval Number 23/2/2018-19/07/2018 and RE/M4H/06/18, respectively. The research procedure was interview-based and minimally invasive (point of care HIV screening test for the control group). Written informed consent was obtained from the caregivers and assent of the child aged seven years and above was obtained. Participants were informed that they could freely withdraw from the study at any time, even after having consented initially, and this did not in any way affect their medical care. In the course of data collection, participants that had significant psychological conditions were counseled and reported to the managing team for possible referral to the mental health unit.

Procedure

The socio-demographic information of the child was obtained by interviewing the child and/or caregiver; information obtained included age, gender, enrollment in school, awareness of the medical condition necessitating hospital visits, drug intake, and survival status of parents. The socio-demographic and clinical information of the child was extracted from the case note and recorded in the data collection form. Social class was determined using the social classification model by Oyedeji, based on the mean score of educational attainment and occupation of both parents [23]. Participants were classified into upper and lower socioeconomic classes based on their mean scores; those that had mean scores of one, two, and three were classified as the upper class while those with mean scores of four and five were classified as lower class. The HIV-related information obtained included a diagnosis of HIV, duration of HIV infection, information on the drug regimen, duration of antiretroviral therapy, number of appointments missed in the past one year, CD4 count, and viral load in the last six months. These were extracted from the case notes by the research assistant and recorded in the case records form.

Clinical staging using the World Health Organization (WHO) Case Definition for HIV Surveillance and clinical staging in children [24] was done by taking a history and doing a general examination. This was performed by the lead researcher who was a senior registrar in the department of pediatrics at the Federal Teaching Hospital Abakaliki, at the time. The immunologic staging was done using the absolute cluster of differentiation 4 (CD4) count according to the WHO immunologic classification for established HIV infection [24]. The latest CD4 count was obtained from the case records and used to classify the immunologic stage of the disease into not significant (CD4 count >500), mild (CD4 count 350-499), advanced (CD4 count 200-349), and severe (CD4 count < 200) [24].

Nutritional status was assessed using the body mass index (BMI) Z-score. The BMI was calculated for each participant using the formula: Weight in kilograms (kg)/Height in meters (m)^2^. Each BMI value was converted to BMI standard deviation (SD) score (Z-score) for age and gender using the WHO 2007 reference growth charts [25] and was then used to grade their nutritional status. The participants were then categorized as severe thinness if <-3SD, thinness if <-2SD, normal if BMI falls between -2SD and +1SD, overweight if >+1SD and obesity if >+2SD [25]. 

The Health-Related Quality of Life (HRQoL) was assessed using the Paediatric Quality of Life Inventory (PedsQL 4.0) [26]. The scale consists of a parallel child report (ages five to 18 years) and a parent proxy report (ages two-18 years). It has an administration time of four minutes and a recall period of four weeks. The PedsQL 4.0 was found to have adequate reliability with internal consistency reliability α-coefficients ranging from 0.83-0.88 for parent proxy report and 0.71-0.84 for child self-report [6]. The validity of the instrument was demonstrated through factor analysis which showed that items on the scale measured the four domains of health (physical, emotional, social, and school functioning) only, as stated by the developers of the instrument. The PedsQL 4.0 was translated to the Igbo language after permission for translation and use of the PedsQL 4.0 was sought and obtained from Mapi Research Trust, on behalf of the authors of the questionnaires. Linguistic validation of the questionnaires was done as stipulated by the Mapi Research Trust and included forward translation step, backward translation, and patient testing. The PedsQL 4.0 consists of a self-report and a proxy report of 23 items each. It measures core domains in physical (eight items), emotional (five items), social (five items), and school (five items) functioning. Items were scored on a five-point Likert scale from zero (Never) to four (Almost always). Scores generated were reverse scored and linearly transformed to a 0-100 scale: 0 = 100, 1 = 75, 2 = 50, 3 = 25 and 4 = 0. The mean domain scale scores were determined by computing the sum of the items over the number of items answered in each domain. If more than 50% of the items on the scale were missing or unanswered the scale score was not computed. The Psychosocial Health Summary Score (15 items) was obtained by summing the items answered in the emotional, social, and school functioning scales, divided by the number of items answered in these domains. The total score was obtained from the sum of the physical and psychosocial health functioning scores divided by the number of items answered in these domains. There are no cut-off points indicating different levels of HRQoL, higher scores indicate better HRQoL. The PedsQL 4.0 consists of developmentally appropriate questionnaires for various age ranges, the questionnaires for 5-7, 8-12, and 13-18 years old were used for this study. Caregivers and children aged 5-7 years old responded to the questionnaires together while caregivers and children aged eight years and above responded independently to the questionnaire. 

The PedsQL 4.0 self-report questionnaire was administered to HIV-positive children and adolescents (cases) and HIV-negative children and adolescents (controls) aged five to 18 years old, and the proxy-report questionnaires were administered to the caregivers of the cases and controls in either English or Igbo language as preferred by the child and caregiver. Some of the questions were modified to suit our local setting, e.g. question six in the physical functioning domain: doing chores like picking up toys was modified to picking up their schoolbooks and clothes as most of the children did not have toys. Age-appropriate containers for fetching water were used to illustrate heavy objects (question four in the physical functioning domain). It took about five minutes to complete the questionnaire.

The caregivers’ burden was assessed using the Zarit Burden Interview (ZBI), which consists of 22 items that measure core domains of personal strain (12 items-questions 1, 4, 5, 8, 9, 14, 16, 17, 18, 19, 20, 21) and role strain (six items-questions 2, 3, 6, 11, 12, 13) [27]. Each domain score was obtained by summing the items in that domain while the total score was obtained by summing all the items. Higher scores indicate a greater caregiver burden. In 2000, Hebert et al. demonstrated its validity, reliability, and internal consistency with Cronbach’s alpha scores of up to 0.92 [28]. It was found suitable for use in a variety of populations of caregivers as scores on this scale were found to be unrelated to the age, gender, locale, language, living situation, marital status, or employment status of the caregivers. The ZBI was administered to caregivers of cases and controls in either English or Igbo language as preferred by the caregiver. It took about 10 minutes to administer the ZBI.

Data analysis

The data generated was sorted, coded, and analyzed using the International Business Machine-Statistical Package of Social Sciences (IBM-SPSS), version 25.0 (IBM Corp., Armonk, NY). The comparison of HRQoL in the subjects and the control group was done using an independent t-test and the effect size and confidence interval were calculated for the domains with a significant difference. The comparison of the nutritional status, clinical and immunologic staging, and HRQoL in the subjects were done using Analysis of variance with Bonferroni posthoc pairwise comparison. Multivariate linear regression was used to determine the predictors of health-related quality of life in the study participants.

## Results

Subjects and the control group were similar in age and gender with a probability value (p-value) of 1.00. However, the two groups differed significantly in their socio-economic status (p-value < 0.001), orphan status (p-value < 0.001), and living circumstances (p-value < 0.001) (Table 1).

**Table 1:** Socio-demographic and nutritional profile of subjects and the control t = Independent t-test, FT = Fischer's Exact Test

Variables	Subjects (n=137)	Control (n=137)	Test stat	p-value
Mean Age (years)	10.67±3.49	10.67±3.49	t=0.00	1.00
Gender			χ^2^=0.00	1.00
Male	64(46.7%)	64(46.7%)		
Female	73(53.3%)	73(53.3%)		
Education			χ^2^=6.10	0.06
Pre-primary	12(8.8%)	11(8.0%)		
Primary	90(65.7%)	72(52.6%)		
Post-primary	35(25.5%)	54(39.4%)		
Social class			χ^2^=86.57	<0.001
Upper	58(42.3%)	111(81.0%)		
Lower	79(57.7%)	26(19.0%)		
Orphan status			FT	<0.001
Double	21(15.3%)	0(0.0%)		
Single (mother)	17(12.4%)	0(0.0%)		
Single (father)	34(24.8%)	4(2.9%)		
None	65(47.5%)	133(97.1%)		
Living status			FT	<0.001
With parents	106(77.4%)	129(94.2%)		
Relatives	27(19.7%)	8(5.8%)		
Non-relatives	4(2.9%)	0(0.0%)		
Nutritional status			FT	0.16
Severe thinness	5(3.6%)	3(2.2%)		
Thinness	12(8.8%)	4(2.9%)		
Normal	107(78.1%)	112(81.8%)		
Overweight	11(8.0%)	12(8.8%)		
Obese	2(1.5%)	6(4.4%)		

Table 2 shows the comparison of HRQoL in the subjects and the control group. Health-related quality of life was significantly lower in the subjects when compared with the controls in the following domains: school functioning (p < 0.001) and psychosocial functioning (p = 0.008). However, there was no significant difference in the overall HRQoL scores.

**Table 2:** Comparison of HRQoL (self-report) between subjects and controls CI=confidence interval; HRQoL=Health-related quality of life

Domains of HRQoL	Subjects (n=137)	Controls (n=137)	t-stat	p-value	Effect size (95%CI)
	Self-reported quality of life				
Physical functioning	94.62±13.39	93.54±6.30	0.848	0.398	-
Emotional functioning	88.72±18.32	87.63±11.73	0.589	0.556	-
Social functioning	97.59±10.20	97.01±5.77	0.583	0.560	-
School functioning	76.75±16.57	87.55±13.68	-5.885	<0.001	-0.69(-2.93 - 1.56)
Psychosocial functioning	87.69±11.24	90.73±7.17	-2.670	0.008	-0.24(-1.72 - 1.23)
Overall health-related quality of life	90.10±11.07	91.71±5.87	-1.505	0.134	-
	Proxy-reported quality of life				
Physical functioning	96.65±7.31	95.55±6.67	1.301	0.195	-
Emotional functioning	92.19±13.56	90.40±12.22	1.147	0.252	-
Social functioning	98.76±5.84	98.87±4.37	-0.176	0.861	-
School functioning	78.91±17.20	88.69±16.86	-4.753	<0.001	-0.75(-2.30 - 0.81)
Psychosocial functioning	89.95±8.95	92.65±8.67	-2.534	0.012	-0.20(-1.79 – 1.39)
Overall health-related quality of life	92.28±7.24	93.67±6.82	-1.633	0.104	-

The association between age, gender, social class, nutritional status and disease severity, and the overall quality of life of the subjects is shown in Table 3. The table shows that these variables (age, gender, social class, nutritional status, and disease severity) were significantly associated with the total score in the health-related quality of life among the cases. 

**Table 3:** The association of age, gender, social class, nutritional status and disease severity with the self-reported health-related quality of life of the cases SD = Standard Deviation, t* = Independent t-test, F = Analysis of Variance; HRQoL=Health-related quality of life

Variables	HR-QoL Total Score for the Child Mean ± SD	t*/F p-value
Age (years)		11.65 <0.001
5-7	94.27±4.62	
8-12	92.13±5.54	
13-18	83.67±16.96	
Gender		-2.04 * 0.04
Male	85.56 ± 18.95	
Female	90.85 ± 8.83	
Social Class		-2.27* 0.025
Upper	87.53 ± 14.69	
Lower	91.84 ± 7.05	
Nutritional Status		5.46 <0.001
Severe Thinness	92.66 ± 4.14	
Thinness	81.18 ± 25.43	
Normal	91.40 ± 6.42	
Overweight	91.49 ± 6.54	
Obese	92.39 ± 4.07	
WHO Clinical Staging		16.80 <0.001
Stage 1	91.86 ± 6.35	
Stage 2	74.46 ± 23.77	
Stage 3	61.96 ± 44.58	
Stage 4	77.17 ± 0.00	
Immunological Staging		20.19 <0.001
Not Significant	91.85 ± 6.1	
Mild	90.54 ± 6.56	
Advanced	89.33 ± 10.23	
Severe	63.66 ± 31.35	

Among the HIV-positive children, the significant predictors of HRQoL were socio-economic status (β-coefficient = 0.19; t = 2.23, p = 0.03), gender (β-coefficient = 0.25; t = 2.83, p = 0.006), caregiver burden score (β-coefficient = -0.21; t = -2.47, p = 0.02), and disease severity (β-coefficient = -0.50; t = -5.67, p < 0.001) (Table 4).

**Table 4:** Predictors of self-reported overall HRQol of life in the subjects Dependent variable = School functioning domain of HRQoL (health-related quality of life) Coefficient of multiple determination (R^2^) = 0.510 β-coefficient=Beta coefficient

Predictors	Beta standardized coefficients	t-stat	p-value
Social class	0.19	2.23	0.03
Age	-0.06	-0.83	0.41
Gender	0.25	2.83	0.006
Orphan status	0.07	0.55	0.58
Body mass index z-score (nutritional index)	0.04	0.46	0.65
Caregiver burden (Zarit caregiver burden score)	-0.21	-2.47	0.02
Disease severity (immunological staging)	-0.50	-5.67	<0.001

## Discussion

The main highlights of this comparative cross-sectional study are: (1) the overall health-related quality of life of HIV-infected children and that of the HIV-negative control group were essentially similar. However, there was a significant difference in the school functioning domains and psychosocial functioning domains between the two groups, with HIV-positive children scoring lower in these domains. (2) Amongst HIV-infected children, upper socioeconomic status, male gender, high scores in caregiver burden, and more severe disease were the significant predictors of lower health-related quality of life.

In this study, the finding that the overall health-related quality of life was similar in the HIV-positive children and the healthy control group is consistent with recent reports [4,14]. For example, Aurpibul et al. [4] in Thailand and Cohen et al. [14] in the Netherlands found no difference in the overall HRQoL scores between HIV-infected children and the HIV-negative controls. The similarities in these studies may be explained by the following factors: firstly, the use of similar instruments and methodology; secondly, most of the children in these studies were asymptomatic and on antiretroviral therapy, a trend similar to the index study where only about 2.2% were in immunological stages 3 and 4. This finding also resonates with the impression that modern treatment modalities have a modifying effect on the various disease outcomes including HRQoL. The current study found that HIV-infected children had significantly lower scores in the school and psychosocial domains, a finding similar to that of Cohen et al. [14] in the Netherlands. In the present study and that by Cohen et al. [14], most HIV-positive children missed school to enable them to attend their routine hospital visits. Such missed school attendance significantly contributed to the lower mean scores in the school and psychosocial domains found in these studies. It is worthy of note that the psychosocial domain score is obtained by summing the scores for the emotional, social, and school domains. Hence, a low school domain score is expected to negatively impact the psychosocial domain score. 

With regards to the potential predictors of HRQoL in HIV-positive children, the present study found that upper socio-economic status, male gender, high scores in caregiver burden, and more severe disease were the significant predictors of lower health-related quality of life. There had been conflicting reports about the relationship between social class and HRQoL, while some authors have reported that upper socio-economic class is associated with better quality of life [15,29], others have noted the opposite or no significant association [4,30]. In other words, it appears that the effect of socio-economic status on HRQoL is diverse. While the upper class may be promotive of a better quality of life in some individuals, in others it may worsen their quality of life. This is probably because high social class is associated with an increase in control of resources (power) and higher respect and esteem from others (status). These two attributes of high social status may improve the quality of life of an individual on one hand and on the other hand expose the individual to undue scrutiny which could impact the quality of life negatively.

The relationship between indices of nutritional state and HRQoL in HIV-positive children has been studied [18,19]. The findings of these studies are in consonance with the present study. These studies were unanimous that indices of poor nutrition are associated with lower scores in the quality-of-life domains [18,19]. Less investigated in the literature is the association between HRQOoL and the burden of caregiving. Literature on social support recognized the distinction between available social resources (structural) and the individuals’ subjective perception or assessment of received support (functional) [31]. The former is reported to be protective against distress while the latter is a “buffer” against the adverse outcomes [31]. Previous studies of patients with other chronic medical conditions have reported that higher perceived social support predicted higher function, quality of life, and better integration into the community [32]. Kurihara et al. found that supportive and favorable attitudes among family members and the community contributed to the improved outcomes in chronic health conditions [33]. These studies support the finding in the present study that a high caregiver burden score predicted lower HRQoL. This finding has implications for care. In other words, apart from services to improve the treatment outcomes of HIV-positive children, there is a need for structured interventions to reduce the burden of care on the caregivers.

The disease severity in the index study, as measured by the clinical stage and CD4 count, was found to have impacted negatively on the HRQoL. Thus, with increasing disease severity, there was a reduction in HRQoL. This finding is similar to the earlier observations of Aurpibul et al. [4] in Thailand and Chitah et al. [17] in Zambia. These authors found that disease severity impacted negatively the HRQoL of HIV-infected children. These findings are not entirely surprising as it is expected that severe disease is more likely to lead to in-patient care, several investigations, and a heavy toll on caregivers’ time and finances. These factors may further reduce the quality of life of the patient. However, Gupta et al. [30] in India got contrasting results, with disease severity having no association with the HRQoL.

Limitations

As a result of the cross-sectional nature of this study, a causal relationship between the health-related quality of life (outcome variable) and the socioeconomic status, nutrition, disease severity, and caregiver’s burden (independent variables) could not be established. 

## Conclusions

The findings of this study show that the overall health-related quality of life of HIV-positive children is approximating that of healthy children. This finding gives clinicians some optimism that with adequate treatment, HIV-positive children will have better outcomes not only in morbidity and mortality but in other psychosocial domains such as health-related quality of life. Measures to reduce school absenteeism when accessing HIV care will improve the school functioning and psychosocial domains of health. In addition, the finding on the relationship between caregiver burden and HRQoL underscores the need to focus on family-based interventions to improve the burden of caregiving.
